# Supplementary material for: Comparative multiomics analysis of cell physiological state after culture in a basket bioreactor
Source: Sci Rep. 2022 Nov 23;12:20161. doi: 10.1038/s41598-022-24687-4 (PMC9686226; doi:10.1038/s41598-022-24687-4)
Supplement: Supplementary file 1 — Supplementary Information 1. [file 41598_2022_24687_MOESM1_ESM.zip › raw data/Transcriptomic raw data/6.Enrichment/1.GO/TE1vsTC1/GSEA/TE1/PROTEOLYSIS_INVOLVED_IN_CELLULAR_PROTEIN_CATABOLIC_PROCESS/PROTEOLYSIS_INVOLVED_IN_CELLULAR_PROTEIN_CATABOLIC_PROCESS(GO_0051603).html]

Details for gene set PROTEOLYSIS\_INVOLVED\_IN\_CELLULAR\_PROTEIN\_CATABOLIC\_PROCESS(GO:0051603)[GSEA]


|  || Dataset | readcount.sample |
| Phenotype | sample.cls |
| Upregulated in class | TE1 |
| GeneSet | PROTEOLYSIS\_INVOLVED\_IN\_CELLULAR\_PROTEIN\_CATABOLIC\_PROCESS(GO:0051603) |
| Enrichment Score (ES) | 0.43502533 |
| Normalized Enrichment Score (NES) | 1.8454957 |
| Nominal p-value | 0.0 |
| FDR q-value | 0.19988753 |
| FWER p-Value | 0.494 |
Table: GSEA Results Summary

  

Fig 1: Enrichment plot: PROTEOLYSIS\_INVOLVED\_IN\_CELLULAR\_PROTEIN\_CATABOLIC\_PROCESS(GO:0051603)
  
Profile of the Running ES Score & Positions of GeneSet Members on the Rank Ordered List

  

| PROBE | DESCRIPTION   (from dataset) | GENE SYMBOL | GENE\_TITLE | RANK IN GENE LIST | RANK METRIC SCORE | RUNNING ES | CORE ENRICHMENT || 1 | 103218706 | LOC103218706 |  |  | 541 | Infinity | -0.0240 | Yes |
| 2 | 103214879 | LOC103214879 |  |  | 1075 | 3.386 | 0.0071 | Yes |
| 3 | 103222611 | PSMA8 |  |  | 1086 | 3.333 | 0.0607 | Yes |
| 4 | 103241474 | SIAH2 |  |  | 2473 | 1.868 | 0.0291 | Yes |
| 5 | 119618125 | UCHL3 |  |  | 3318 | 1.641 | 0.0179 | Yes |
| 6 | 103216824 | ATE1 |  |  | 3684 | 1.577 | 0.0272 | Yes |
| 7 | 103217971 | CUL3 |  |  | 3820 | 1.553 | 0.0464 | Yes |
| 8 | 103226162 | PSMA2 |  |  | 3924 | 1.537 | 0.0667 | Yes |
| 9 | 103230396 | UCHL5 |  |  | 4069 | 1.518 | 0.0849 | Yes |
| 10 | 103236978 | ELOC |  |  | 4135 | 1.510 | 0.1065 | Yes |
| 11 | 103219057 | RAD23B |  |  | 4195 | 1.502 | 0.1283 | Yes |
| 12 | 103240980 | PSMB1 |  |  | 4329 | 1.481 | 0.1464 | Yes |
| 13 | 103216597 | CACUL1 |  |  | 4363 | 1.477 | 0.1689 | Yes |
| 14 | 103239807 | PSMA1 |  |  | 4529 | 1.456 | 0.1851 | Yes |
| 15 | 103238047 | LOC103238047 |  |  | 4673 | 1.439 | 0.2021 | Yes |
| 16 | 103227180 | CUL1 |  |  | 4689 | 1.437 | 0.2248 | Yes |
| 17 | 103232443 | SIAH1 |  |  | 4733 | 1.432 | 0.2461 | Yes |
| 18 | 103239889 | PSMB7 |  |  | 5265 | 1.373 | 0.2446 | Yes |
| 19 | 103238109 | CUL2 |  |  | 5413 | 1.358 | 0.2601 | Yes |
| 20 | 103214836 | CUL4A |  |  | 5547 | 1.343 | 0.2759 | Yes |
| 21 | 103243135 | PSMA7 |  |  | 5688 | 1.332 | 0.2913 | Yes |
| 22 | 103244544 | SKP1 |  |  | 5829 | 1.319 | 0.3064 | Yes |
| 23 | 103248397 | CUL5 |  |  | 5879 | 1.313 | 0.3256 | Yes |
| 24 | 103229087 | PSMA3 |  |  | 6735 | 1.240 | 0.3074 | Yes |
| 25 | 103225984 | UBE4B |  |  | 7217 | 1.204 | 0.3055 | Yes |
| 26 | 103246532 | LOC103246532 |  |  | 7257 | 1.200 | 0.3232 | Yes |
| 27 | 103223000 | UFD1 |  |  | 7764 | 1.160 | 0.3194 | Yes |
| 28 | 103242203 | PSMB6 |  |  | 7964 | 1.146 | 0.3291 | Yes |
| 29 | 103218996 | CDC26 |  |  | 8044 | 1.139 | 0.3441 | Yes |
| 30 | 103231459 | PSMB11 |  |  | 8121 | 1.134 | 0.3591 | Yes |
| 31 | 103234004 | RAD23A |  |  | 9084 | 1.080 | 0.3336 | Yes |
| 32 | 103224487 | LOC103224487 |  |  | 9107 | 1.079 | 0.3501 | Yes |
| 33 | 103243561 | PSMB3 |  |  | 9126 | 1.077 | 0.3668 | Yes |
| 34 | 103246177 | UCHL1 |  |  | 9200 | 1.072 | 0.3809 | Yes |
| 35 | 103227728 | BAP1 |  |  | 9377 | 1.061 | 0.3903 | Yes |
| 36 | 103247359 | CUL4B |  |  | 9625 | 1.046 | 0.3962 | Yes |
| 37 | 103245250 | PSMA4 |  |  | 9695 | 1.041 | 0.4100 | Yes |
| 38 | 103231457 | PSMB5 |  |  | 9933 | 1.025 | 0.4160 | Yes |
| 39 | 103224033 | PSMB4 |  |  | 10344 | 1.001 | 0.4139 | Yes |
| 40 | 103221472 | CUL9 |  |  | 10375 | 0.999 | 0.4288 | Yes |
| 41 | 103248589 | UBE4A |  |  | 10626 | 0.982 | 0.4336 | Yes |
| 42 | 103228819 | PSMA6 |  |  | 10943 | 0.960 | 0.4350 | Yes |
| 43 | 103225129 | PSMB2 |  |  | 12475 | 0.864 | 0.3806 | No |
| 44 | 103239578 | ANAPC2 |  |  | 13127 | 0.825 | 0.3648 | No |
| 45 | 103221475 | CUL7 |  |  | 13465 | 0.803 | 0.3628 | No |
| 46 | 103238477 | LOC103238477 |  |  | 17245 | 0.554 | 0.2027 | No |
| 47 | 103221690 | PSMB8 |  |  | 18129 | 0.478 | 0.1710 | No |
| 48 | 103233253 | PSMB10 |  |  | 19266 | 0.364 | 0.1261 | No |
| 49 | 103221693 | PSMB9 |  |  | 19351 | 0.356 | 0.1281 | No |
| 50 | 103241738 | LOC103241738 |  |  | 19595 | 0.329 | 0.1225 | No |
| 51 | 103246089 | LOC103246089 |  |  | 20527 | 0.185 | 0.0839 | No |
Table: GSEA details
[plain text format]

  

Fig 2: PROTEOLYSIS\_INVOLVED\_IN\_CELLULAR\_PROTEIN\_CATABOLIC\_PROCESS(GO:0051603)
  
Blue-Pink O' Gram in the Space of the Analyzed GeneSet

  

Fig 3: PROTEOLYSIS\_INVOLVED\_IN\_CELLULAR\_PROTEIN\_CATABOLIC\_PROCESS(GO:0051603): Random ES distribution
  
Gene set null distribution of ES for
 **PROTEOLYSIS\_INVOLVED\_IN\_CELLULAR\_PROTEIN\_CATABOLIC\_PROCESS(GO:0051603)**
